# Prevalence, Incidence, and External Causes of Traumatic Spinal Cord Injury in China: A Nationally Representative Cross-Sectional Survey

**DOI:** 10.3389/fneur.2021.784647

**Published:** 2022-01-20

**Authors:** Bin Jiang, Dongling Sun, Haixin Sun, Xiaojuan Ru, Hongmei Liu, Siqi Ge, Jie Fu, Wenzhi Wang

**Affiliations:** ^1^Department of Neuroepidemiology, Beijing Neurosurgical Institute, Beijing Tiantan Hospital, Capital Medical University, Beijing, China; ^2^Beijing Municipal Key Laboratory of Clinical Epidemiology, Beijing, China; ^3^National Office for Cerebrovascular Diseases (CVD) Prevention and Control in China, Beijing, China

**Keywords:** prevalence, incidence, external causes, traumatic spinal cord injury, China

## Abstract

**Background and Purpose:**

The epidemiological characteristics of traumatic spinal cord injury (TSCI) in China are unclear. Thus, we aimed to study prevalence, incidence, and external causes of TSCI in China nationwide.

**Methods:**

In 2013, we conducted a nationally representative, door-to-door epidemiological survey on TSCI in China using a complex, multistage, probability sampling design.

**Results:**

In China, the point prevalence of TSCI standardized to the China census population 2010 was 569.7 (95% CI: 514.2–630.4) per 1,000,000 in the population, 753.6 (95% CI: 663.3–854.3) per 1,000,000 among men, and 387.7 (95% CI: 324.8–461.1) per 1,000,000 among women. The incidence of TSCI standardized to the China census population 2010 was 49.8 (95% CI: 34.4–70.7) per 1,000,000 per year in the population, 63.2 (95% CI: 38.9–98.5) per 1,000,000 among men, and 36.9 (95% CI: 19.5–65.9) per 1,000,000 among women. Among the 415 TSCI events in 394 prevalent cases, the top three injury causes were falls (55.2%), motor vehicle collisions (MVCs) (26.5%), and strike injuries (10.1%), while other injury causes including gunshot and explosion accounted for 8.2%. Among the 394 prevalent cases, the mean age of patients at the time of injury was 43.7 ± 17.1 years; the male-to-female ratio was 1.86:1.

**Conclusion:**

It is estimated that there are 759,302 prevalent patients with TSCI in total and 66,374 new TSCI cases annually in China. Falls and MVCs are still 2 major external causes for TSCI in China.

## Introduction

Traumatic spinal cord injury (TSCI) once caused may lead to different degrees of paralysis, loss of sensory, and dysfunction of bladder or bowel. As one of the most devastating kinds of injury, TSCI not only affect one's health, but also generates a huge economic burden on the family and society. Since there is no curative hope for permanent spinal cord injury, prevention of TSCI is particular important (1). In previous studies, the global incidence of TSCI varied from 2.3 (2) to 150.6 (3) cases per million inhabitants per year, whereas the global prevalence varied from 236.0 (2, 4) to 1,800.0 (2) per million inhabitants. In contrast, due to the lack of national level monitoring data, the epidemiological data of TSCI in China are relatively scarce compared with other countries and regions (1, 2, 4). Previous studies are mainly confined to the incidence of TSCI sporadically in Beijing (5), Tianjin (6), Xi'an (7), and Taiwan (3, 8–10) and more focus is on clinical epidemiological investigation on TSCI in Beijing (11), Tianjin (12), Chongqing (13), Guangdong (14–16), Xi'an (7), and Heilongjiang (17). However, so far, not only there is no national representative data on the incidence of TSCI in China nationwide, but also little information is available with respect to the prevalence of TSCI in China. Therefore, we adopted a multistage, complex sampling method to investigate the prevalence and incidence of TSCI in China nationwide, based on the national epidemiological survey of cerebrovascular diseases in China (18, 19).

## Methods

### Sampling Design, Quality Assurance, and Participants

**Figure 1:**
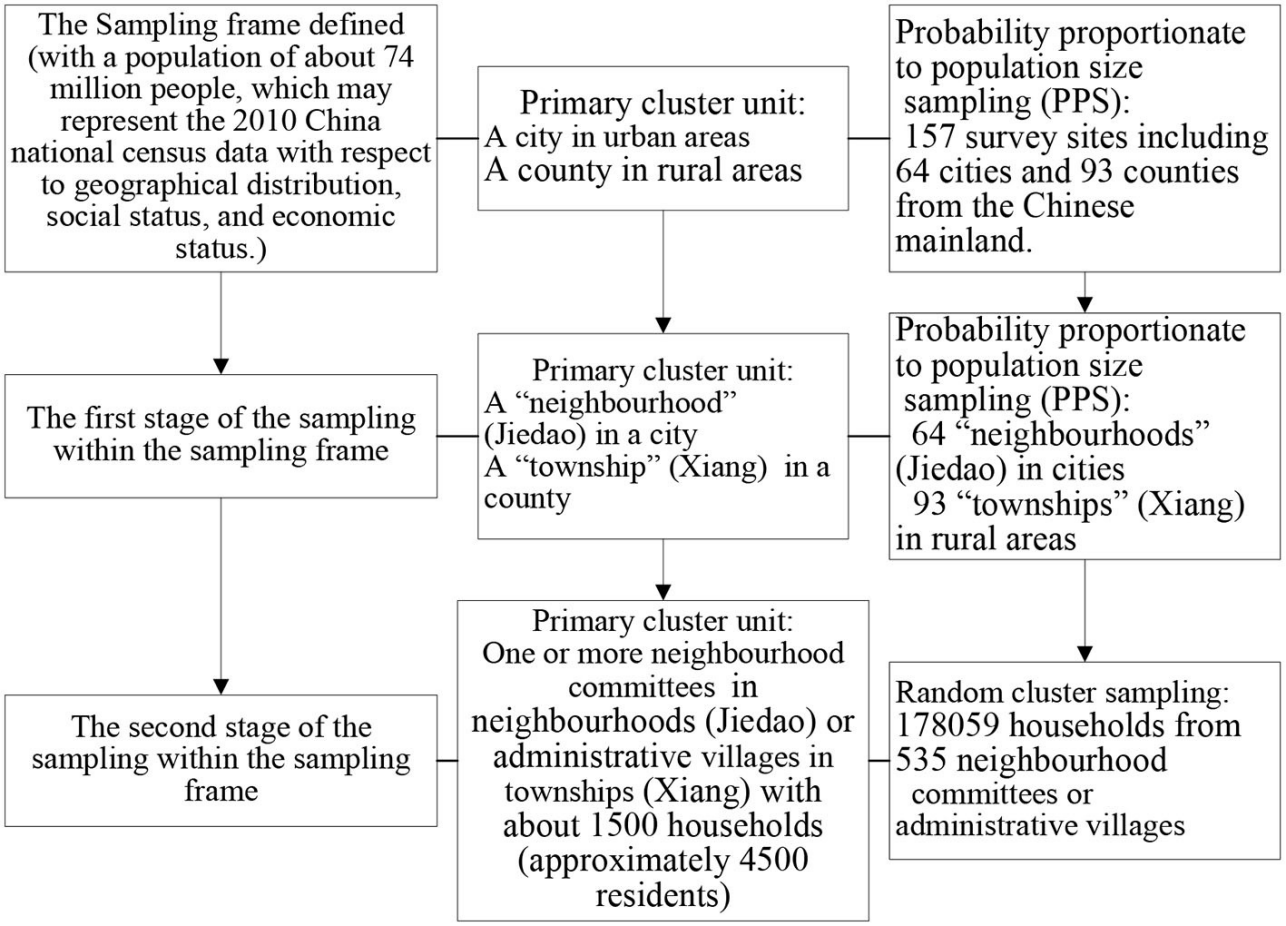
Sampling flowchart.

**Figure 2:**
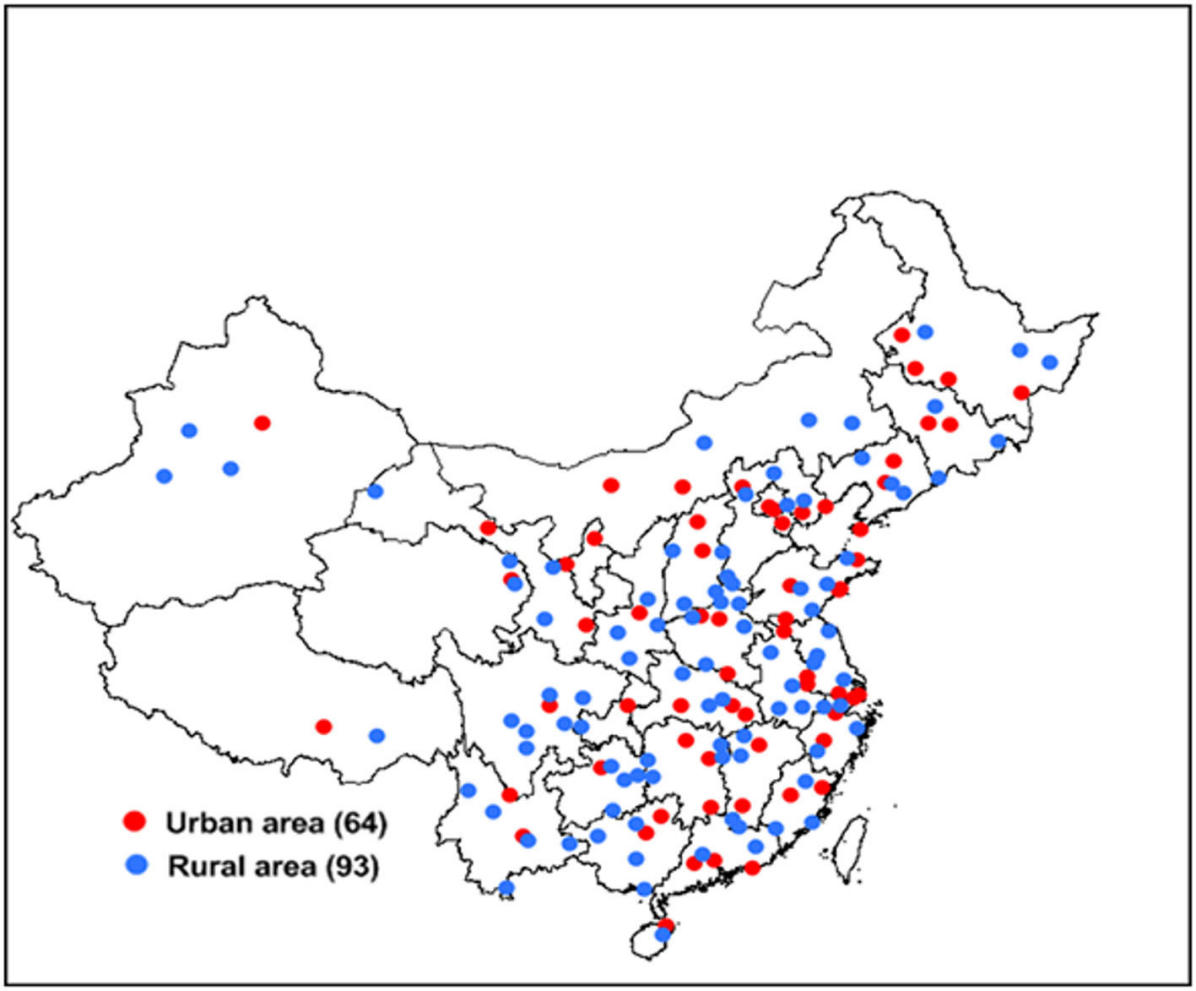
Distribution of survey sites in 31 provinces of China.

The complex, multistage probability sampling design used to define the sampling frame and the participants has been described in detail in previous studies (18–20) (Figure 1). In brief, 2010 Chinese population census data and probability proportionate to population size (PPS) sampling were used to select 64 urban and 93 rural areas from 31 provinces of China [i.e., 157 disease surveillance points (DSPs) or survey sites shown in Figure 2]. In the first stage of sampling, PPS sampling was again used to select “neighborhoods” (Jiedao) within cities or “townships” (Xiang) in rural areas; the probability of selection was based on the population size of the neighborhood or township. In the second stage of sampling, one or more neighborhood committees (administrative villages) with a total population of at least 4,500 residents (~1,500 households) were selected from the sampled neighborhoods (townships) at each site using random cluster sampling.

Detailed quality assurance methods have been described in previous studies (18–20). In brief, quality control was performed in all the phases of the survey and survey preparations, field work, and data processing were all supervised. Trained investigators visited these participants at least 3 times on different dates to ensure the response. Two of 157 DSPs were excluded from the final data analysis due to not meeting the requirements of the study design.

**Figure 3:**
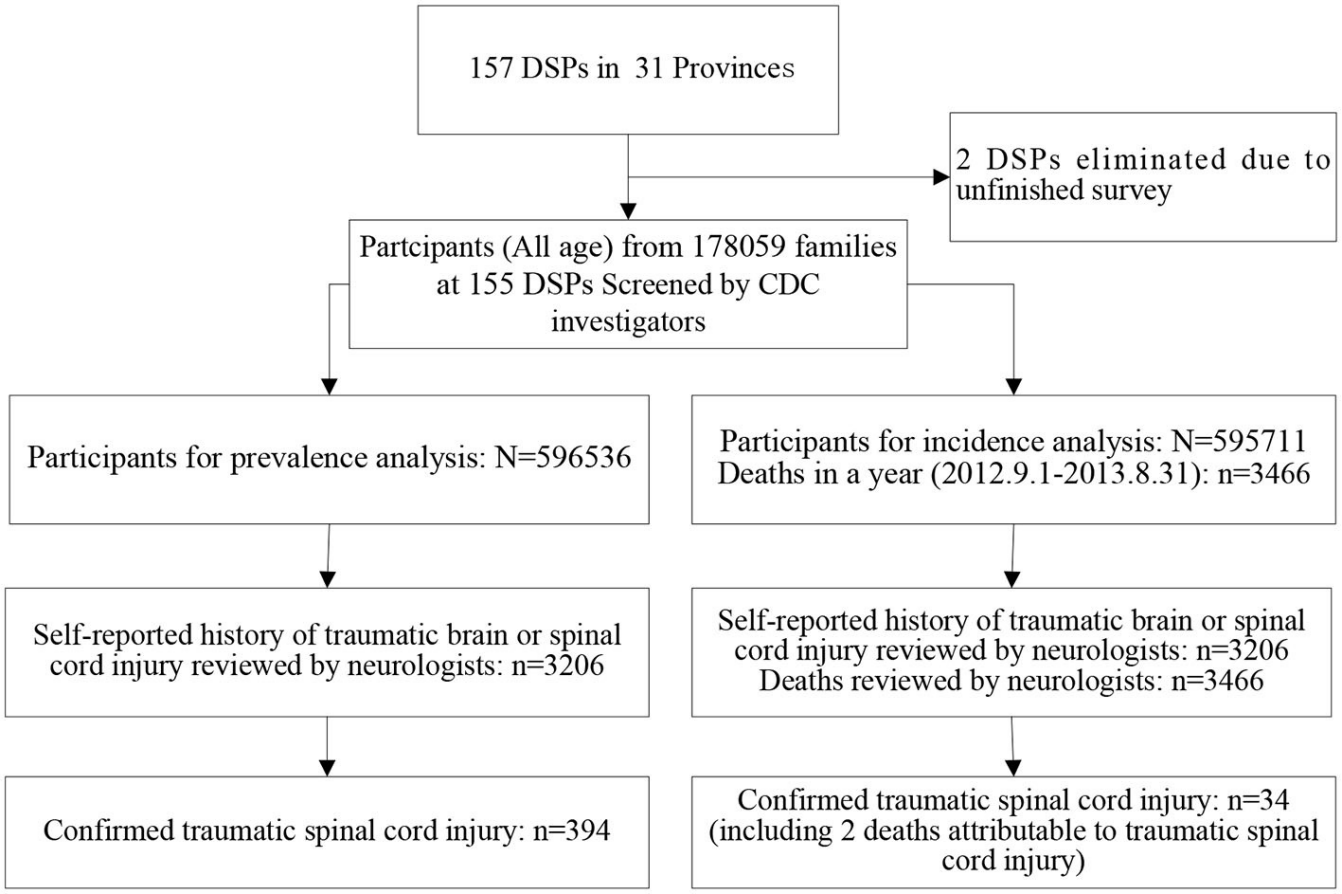
Flowchart for traumatic spinal cord injury (TSCI) case ascertainment. DSPs, disease surveillance points; CDC, Centers for Disease Control and Prevention.

The participants included people who had lived in the county (or district) for at least 6 months in the past year. In this retrospective epidemiological survey, TSCI point prevalence was defined as the rate of patients with TSCI among the survival people prior to midnight on August 31, 2013 from the sampled families. TSCI incidence was defined as the rate of patients with TSCI occurred within a year among the survival population prior to midnight on August 31, 2012 from the sampled families. For prevalence and incidence analyses in this survey, 596,536 and 595,711 people from the 178,059 families were finally used (see Figure 3), respectively, with a response rate of about 81% (19).

### Diagnostic Criteria and Case Ascertainment

In this epidemiological study, a TSCI was defined as the occurrence of an acute lesion on the neural elements in the spinal canal (spinal cord and cauda equina), resulting in temporary or permanent sensory deficits, motor deficits, or bladder/bowel dysfunction (6). Other non-traumatic causes such as degenerative spinal change and surgical damage were excluded. From September 1 to December 31, 2013, the trained Centers for Disease Control and Prevention (CDC) investigators visited each eligible household, collected the signed informed consent forms of participants, and administered a structured questionnaire for TSCI data. The information on demographic characteristics including age, gender, education, occupation, and medical history of the individuals and data on times and dates, sites, symptoms and signs, external cause, and medical treatments of TSCI were also obtained and reviewed. Self-reported history of traumatic brain or spinal cord injury was further reviewed by our neurological reviewers. The validated verbal autopsy technique involving household members of people who died within the 12 months preceding the survey was used to identify TSCI as a possible cause of death.

### Statistical Analysis

In contrast to higher crude incidence in men than that in women in this survey, higher weighted incidence in women than that in men disapproved of using weighting during the analysis, although weighting is usually used to account for the complex sampling designs.

Sociodemographic characteristics of the study sample were categorized and presented as frequency and percent. Crude prevalence and incidence of TSCI were calculated by subgroups of age (0–14, 15–24, 25–34, 35–44, 45–54, 55–64, 65–74, and ≥ 75 years), sex (men/women), place of residence (urban/rural), and geographic location (eastern/central/western China). For comparison, the prevalence and incidence of overall age groups were directly standardized to the age distribution of the WHO world standard population and the China census population 2010, respectively. The 95% CIs for all the crude and age-standardized rates were also calculated. Prevalent and incident numbers of TISC in China nationwide were estimated based on the age-standardized rates of the China census population 2010. Furthermore, a Poisson regression analysis was used to compare the rate ratio of the prevalence and incidence of TSCI among different subgroups of population in China, 2013. Age group, sex, place of residence, and geographic location were adjusted each other in all the Poisson regression analyses. The prognosis for TSCI in the population was estimated based on the prevalence and incidence of TSCI in the population, which was in fact a rate ratio of prevalence to incidence in the population different from the prognosis estimates in a cohort (19). Given that the number of incident cases was too small, the external causes and risky occupations for TSCI are also analyzed by prevalent cases. The comparison of rates between the different groups was performed by the chi-squared test. All of these statistical calculations on complex samples were performed using the SPSS version 15.0 software (SPSS Incorporation, Chicago, Illinois, USA). *p* < 0.05 was considered as statistically significant.

## Results

The characteristics of the study sample from the national epidemiological survey of TSCI in China, 2013 are shown in Table 1. Among the 596,536 people evaluated for the prevalence analysis, 394 survival TSCI cases were identified on August 31, 2013 (see Table 2, Figure 3, and Supplementary Table 1). Among the 394 cases, 66.7% were confirmed with CT/MRI imaging; the mean age of patients at the time of injury was 43.7 ± 17.1 years; the male-to-female ratio was 1.86:1; there was 299 isolated TSCI events and 116 concomitant TSCI and traumatic brain injury (TBI) in total. Among the 595,711 people assessed for the incidence analysis, 34 TSCI cases (including 2 deaths) were found between September 1, 2012 and August 31, 2013 (see Table 3; Figure 3). Among the 34 cases, the mean age of patients at the time of injury was 56.0 ± 17.0 years; the male-to-female ratio was 1.62:1.

**Table 1:** Characteristics of the study sample of the national epidemiological survey of traumatic spinal cord injury (TSCI) in China, 2013.

	**Prevalence**		**Incidence**	
**Characteristics**	**No**.	**%**	**No**.	**%**
**Age group**				
0~	83,028	13.9%	85,462	14.3%
15~	77,354	13.0%	81,378	13.7%
25~	91,435	15.3%	89,597	15.0%
35~	99,582	16.7%	102,999	17.3%
45~	93,763	15.7%	90,667	15.2%
55~	80,155	13.4%	78,075	13.1%
65~	44,840	7.5%	43,245	7.3%
75~	26,379	4.4%	24,288	4.1%
**Sex**				
Men	30,0192	50.3%	299,725	50.3%
Women	296,344	49.7%	295,986	49.7%
**Education**				
Primary school or preschool	248,916	41.7%	245,192	41.1%
Middle school	294,209	49.3%	294,193	49.4%
College and higher	51,730	8.7%	51,721	8.7%
Unknown	1,681	0.3%	4,605	0.8%
**Occupation**				
Student	108,978	18.3%	105,510	17.7%
Worker	45,021	7.5%	45,004	7.6%
Farmer or farmer worker	271,068	45.4%	270,916	45.5%
Employee	46,676	7.8%	46,674	7.8%
Self-employed	52,518	8.8%	52,516	8.8%
Retiree or homemaker	66,169	11.1%	66,145	11.1%
other	4,439	0.7%	4,356	0.7%
Unknown	1,667	0.3%	4,590	0.8%
**Place of residence**				
Urban	282,945	47.4%	282,169	47.4%
Rural	313,591	52.6%	313,542	52.6%
**Geographic location**				
Eastern China	201,354	33.8%	201,196	33.8%
Central China	239,735	40.2%	239,288	40.2%
Western China	155,447	26.1%	155,227	26.1%

**Table 2:** Prevalence^*^ of TSCI in China, 2013 (1/1,000,000 person × life time).

	**Men**				**Women**				**Total**	
**Age group**	**Population**	**Cases**	Prevalence^*^	95% CI^*^	**Population**	**Cases**	Prevalence^*^	95% CI^*^	Prevalence^*^	95% CI^*^
0~	443,64	1	22.5	0.6–125.6	38,664	1	25.9	0.7–144.1	24.1	2.9–87.0
15~	39,589	6	151.6	55.6–329.9	37,765	2	53.0	6.4–191.3	103.4	44.6–203.8
25~	45,020	21	466.5	288.7–713.0	46,415	9	193.9	88.7–368.1	328.1	221.4–468.4
35~	50,759	37	728.9	513.2–1,004.7	48,823	15	307.2	172.0–506.7	522.2	390.0–684.8
45~	46,879	62	1,322.6	1,014.0–1,695.5	46,884	28	597.2	396.8–863.1	959.9	771.8–1,179.8
55~	39,366	62	1,575.0	1,207.5–2,019.0	40,789	42	1,029.7	742.1–1,391.8	1,297.5	1,060.1–1,572.1
65~	21,902	49	2,237.2	1,655.1–2,957.7	22,938	23	1,002.7	635.6–1,504.5	1,605.7	1,256.4–2,022.1
75~	12,313	18	1,461.9	866.4–2,310.4	14,066	18	1,279.7	758.4–2,022.4	1,364.7	955.8–1,889.4
Total	300,192	256	852.8	751.5–963.9	296,344	138	465.7	391.2–550.2	660.5	596.9–729.0
Age-adjusted rates^#^	–	–	645.4	567.4–735.1	–	–	332.2	277.7–400.0	487.9	439.9–541.9
Age-adjusted rates^$^	–	–	753.6	663.3–854.3	–	–	387.7	324.8–461.1	569.7	514.2–630.4

* *A point prevalence in a life time, on August 31, 2013*.

# *Age standardized to the WHO world standard population*.

$ *Age standardized to the China census population 2010*.

**Table 3:** Incidence^*^ of TSCI in China, 2013 (1/1,000,000 person × years).

	**Men**				**Women**				**Total**	
**Age group**	**Population**	**Cases**	Incidence^*^	95% CI^*^	**Population**	**Cases**	Incidence^*^	95% CI^*^	Incidence^*^	95% CI^*^
0~	45,548	0	–	–	39,914	1	25.1	0.6–139.6	11.7	0.3–65.2
15~	41,209	1	24.3	0.6–135.2	40,169	0	–	–	12.3	0.3–68.5
25~	44,351	1	22.6	0.6–125.6	45,246	1	22.1	0.6–123.1	22.3	2.7–80.6
35~	52,561	3	57.1	11.8–166.8	50,438	1	19.8	0.5–110.5	38.8	10.6–99.4
45~	45,289	4	88.3	24.1–226.1	45,378	0	–	–	44.1	12.0–113.0
55~	38,189	7	183.3	73.7–377.7	39,886	5	125.4	40.7–292.5	153.7	79.4–268.5
65~	21,283	3	141.0	29.1–411.9	21,962	4	182.1	49.6–466.3	161.9	65.1–333.5
75~	11,295	2	177.1	21.4–639.6	12,993	1	77.0	1.9–428.8	123.5	25.5–361.0
Total	299,725	21	70.1	43.4–107.1	295,986	13	43.9	23.4–75.1	57.1	39.5–79.6
Age-adjusted rates^#^	–	–	53.3	32.7–87.5	–	–	34.9	17.3–69.1	43.8	29.8–64.7
Age-adjusted rates^$^	–	–	63.2	38.9–98.5	–	–	36.9	19.5–65.9	49.8	34.4–70.7

* *Annual incidence between September 1, 2012 and August 31, 2013*.

# *Age standardized to the WHO world standard population*.

$ *Age standardized to the China census population 2010*.

### Prevalence of TSCI

**Figure 4:**
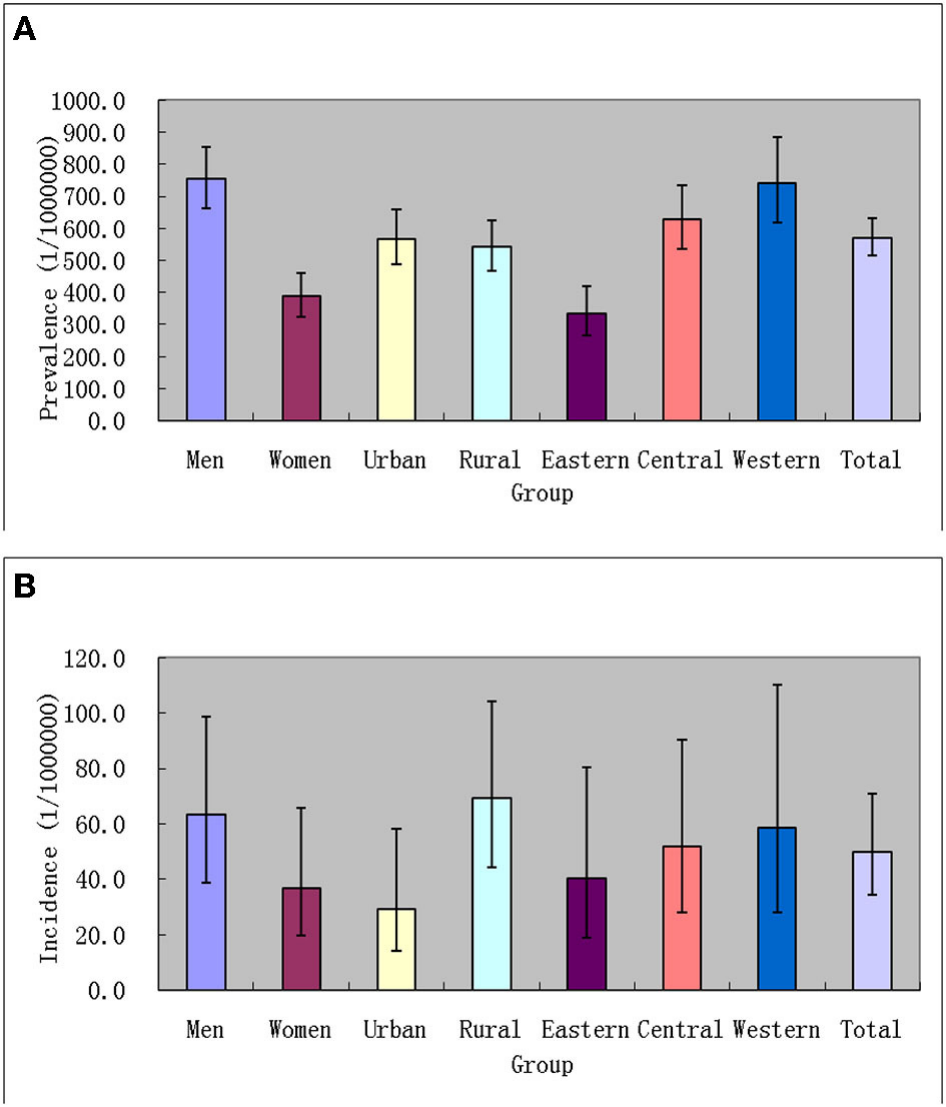
Prevalence [**(A)** 1/1,000,000 person × life time] and incidence [**(B)** 1/1,000,000 person × years] of TSCI in China, 2013.

In China, the point prevalence of TSCI standardized to the China census population 2010 was 569.7 (95% CI: 514.2–630.4) per 1,000,000 in the population, 753.6 (95% CI: 663.3–854.3) per 1,000,000 among men, and 387.7 (95% CI: 324.8–461.1) per 1,000,000 among women; 567.7 (95% CI: 489.1–658.1) per 1,000,000 among urban residents and 541.3 (95% CI: 466.3–625.8) per 1,000,000 among rural residents; 335.4 (95% CI: 267.0–420.1) per 1,000,000 among eastern Chinese, 627.8 (95% CI: 536.6–732.5) per 1,000,000 among central Chinese, and 741.6 (95% CI: 616.4–886.1) per 1,000,000 among western Chinese (see Table 2; Figure 4). According to the above-estimated prevalence, there were an estimated 759,302 (95% CI: 685,331–840,204) patients with TSCI in the population with 514,203 (95% CI: 452,589–582,914) male patients with TSCI and 252,192 (95% CI: 211,277–299,937) female patients with TSCI in China.

After adjusting for other factors, the prevalence of TSCI increased with age (see Table 4). The prevalence of TSCI among men was significantly higher than that among women (rate ratio: 1.904; 95% CI: 1.548–2.342; see Table 4). The prevalence of TSCI among eastern Chinese was significantly lower than that among western Chinese (rate ratio: 0.462; 95% CI: 0.352–0.606; see Table 4). No difference in prevalence of TSCI was found between the urban and rural residents (see Table 4).

**Table 4:** Prevalence (1/1,000,000 person × life time), incidence (1/1,000,000 person × years), and rate ratio of TSCI among different subgroups of population in China, 2013.

	**Prevalence**			**Incidence**		
**Factors**	**Rate (95%CI)**	**Rate ratio^#^** **(95%CI)**	***P* value**	**Rate (95%CI)**	**Rate ratio^#^** **(95%CI)**	***P* value**
**Age group**						
0~	24.1 (2.9–87.0)	0.016 (0.004–0.067)	<0.001	11.7 (0.3–65.2)	0.084 (0.009–0.808)	0.032
15~	103.4 (44.6–203.8)	0.069 (0.032–0.149)	<0.001	12.3 (0.3–68.5)	0.087 (0.009–0.842)	0.035
25~	328.1 (221.4–468.4)	0.229 (0.141–0.372)	<0.001	22.3 (2.7–80.6)	0.170 (0.028–1.017)	0.052
35~	522.2 (390.0–684.8)	0.356 (0.232–0.544)	<0.001	38.8 (10.6–99.4)	0.292 (0.065–1.309)	0.108
45~	959.9 (771.8–1,179.8)	0.672 (0.456–0.989)	0.044	44.1 (12.0–113.0)	0.335 (0.075–1.496)	0.152
55~	1,297.5 (1,060.1–1,572.1)	0.934 (0.640–1.365)	0.725	153.7 (79.4–268. 5)	1.179 (0.333–4.179)	0.799
65~	1,605.7 (1,256.4–2,022.1)	1.125 (0.754–1.679)	0.563	161.9 (65.1–333.5)	1.257 (0.325–4.865)	0.741
75~	1,364.7 (955.8–1,889.4)	Reference		123.5 (25.5–361.0)	Reference	
**Sex**						
Men	852.8 (751.5–963.9)	1.904 (1.548–2.342)	<0.001	70.1 (43.4–107.1)	1.640 (0.821–3.277)	0.161
Women	465.7 (391.2–550.2)	Reference		43.9 (23.4–75.1)	Reference	
**Place of residence**						
Urban	682.1 (589.3–785.4)	1.060 (0.868–1.294)	0.565	35.4 (17.0–65.2)	0.438 (0.208–0.921)	0.029
Rural	605.9 (522.8–698.4)	Reference		76.5 (49.0–113.9)	Reference	
**Geographic location**						
Eastern China	427.1 (341.6–527.5)	0.462 (0.352–0.606)	<0.001	49.7 (23.8–91.4)	0.708 (0.294–1.704)	0.441
Central China	721.6 (618.1–837.5)	0.816 (0.649–1.026)	0.082	58.5 (32.0–98.2)	0.937 (0.414–2.121)	0.876
Western China	797.7 (663.5–951.1)	Reference		64.4 (30.9–118.5)	Reference	

# *Age group, sex, place of residence, and geographic location were adjusted each other in all the Poisson regression analyses*.

### Incidence of TSCI

In China, the incidence of TSCI standardized to the China census population 2010 was 49.8 (95% CI: 34.4–70.7) per 1,000,000 per year in the population, 63.2 (95% CI: 38.9–98.5) per 1,000,000 among men, and 36.9 (95% CI: 19.5–65.9) per 1,000,000 among women; 29.4 (95% CI: 14.0–58.1) per 1,000,000 among urban residents and 69.2 (95% CI: 44.2–104.3) per 1,000,000 among rural residents; 40.4 (95% CI: 18.9–80.3) per 1,000,000 among eastern Chinese, 52.0 (95% CI: 28.2–90.4) per 1,000,000 among central Chinese, and 58.8 (95% CI: 28.1–110.1) per 1,000,000 among western Chinese (see Table 3; Figure 4). According to the above-estimated incidence, there were an estimated 66,374 (95% CI: 45,849–94,230) patients with TSCI annually in the population, with 43,123 (95% CI: 26,543–67,209) male patients with TSCI and 24,003 (95% CI: 12,684–42,867) female patients with TSCI in China.

After adjusting for other factors, the incidence of TSCI increased with age (see Table 4). The incidence of TSCI among urban residents was significantly lower than that among rural residents (rate ratio: 0.438; 95% CI: 0.208–0.921; see Table 4). No difference in incidence of TSCI was found between different subgroups of sex and geographic location (see Table 4).

### Prognosis for TSCI in the Population

In China, the average prognosis for TSCI in the population was estimated to be 11.57 (95% CI: 8.15–16.43) years based on estimates of point prevalence in a lifetime and the annual incidence of TSCI.

### External Cause and Risky Occupation for TSCI

Among the 415 TSCI events, the top three injury causes were falls (55.2%), motor vehicle collisions (MVCs) (26.5%), and strike injuries (10.1%), while other injury causes including gunshot and explosion accounted for 8.2%. The consistent injury causes (i.e., 64.7% for falls, 23.5% for MVCs, and 11.8% for strike injuries) were found in the 34 incident cases of TSCI. No difference in external cause was found between the 2 groups χ^2^ = 3.484; *p* = 0.323.

Among the 394 prevalent cases, the top four injury occupations were farmer or migrant workers from the villages (61.2%), retiree or homemaker (19.5%), the self-employed (9.9%), and worker (4.6%), while other classifications of occupation including employee or students accounted for 4.8%. The consistent risky occupation was found in the 34 incident cases of TSCI (data not shown). No difference in occupational risk was found between the 2 groups (χ^2^ = 4.990; *p* = 0.288). Among the 394 cases, primary school, middle school, college and higher, and preschool accounted for 49.2, 46.2, 3.3, and 1.3%, respectively.

## Discussion

### Prevalence

**Figure 5:**
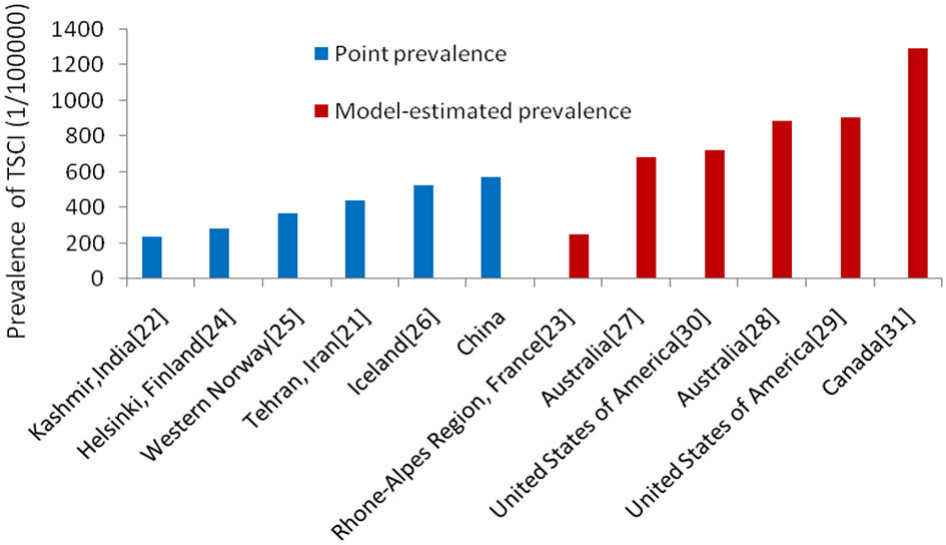
Point prevalence (blue) and model-estimated prevalence (red) of TSCI from different regions and countries.

Supplementary Table 2 lists the study design, case definition, and findings of previous prevalence surveys of TSCI in different regions or countries (21–31). The prevalence of SCI was highest in the USA (1,800 per million population) (2) and lowest in Kashmir, India (236 per million population) (22). Obviously, different study designs and definitions have a great impact on the study results. According to the design, there are roughly two types: one is the cross-sectional point prevalence survey (21, 22, 24–26); the other is based on the estimation of incidence rate and duration of TSCI or other more complex estimation (23, 27–31). Except for individual model estimate (23), it seems that the prevalence estimated by models (27–31) is generally higher than the point prevalence in the cross-sectional surveys (21, 22, 24–26). In this survey, the prevalence of TSCI is higher than that in other cross-sectional surveys (21, 22, 24–26) and estimated by a model in a previous study (23), but lower than that estimated by a model in most studies (27–31) (Figure 5). In this survey, the average prognosis for TSCI in the population was estimated to be 11.57 years based on the point prevalence and annual incidence of TSCI. Obviously, the average prognosis in China was lower than the average life durations of about 20 years (23) and 40.35 years (27) adopted for prevalence estimations in previous studies. It is worth noting that most previous studies did not give a clear definition of TSCI (21–23, 27–31). Moreover, the ICD codes for TSCI given by two previous studies are also different (24, 26). Indeed, the prevalence of TSCI in this survey was higher than that in a previous study (25), although same design and definition of TSCI adopted in both the studies.

### Incidence

Supplementary Table 3 shows that the incidence of TSCI was significantly different across different countries, regions, and cities (3, 5–10, 21, 26, 32–48). This could be a reflection of actual differences in incidence or a result of differences in case ascertainment. For example, some studies have used information from death certificates, coroners, or the department of legal medicine to include TSCI victims who have died at the scene of the accident or during transport to acute care centers (32, 33, 40, 45). Other studies have excluded these patients from their estimates. In addition, identification of patients with acute SCI was done in different ways across studies. Some used ICD-9 or ICD-10 codes to detect relevant patients (3, 6, 7, 10, 26, 32–34, 40, 41, 44, 45), whereas others used a simple clinical definition, surveys, or questionnaires (5, 8, 10, 21, 35–39, 41, 43, 46–48). The low incidence of SCI from some countries may not be accurate, since data may be aggregated from hospitals or rehabilitation centers and not directly collected (49). In order to make comparisons between countries or to accurately estimate national or regional incidence, methodologies of data collection must be standardized (50). According to previous reports, the incidence rate of TSCI worldwide is 2.3 (2) to 150.6 (3) per million per year. In this survey, the incidence of TSCI was 49.8 per million per year in China, which was higher than that in most previous findings (6–8, 10, 21, 26, 35–44, 46, 47), but lower than that in other studies (3, 5, 9, 32–34, 45, 48). The incidence of TSCI in this survey increased with age before 75 years old. The incidence of TSCI reached a peak in the 65–74-year-old group. Previous studies have shown that the age of patients with SCI trends to be bimodal distribution, the first peak is 15–29 years, and the second peak is over 65 years (51). However, the first peak did not occur in this survey, probably attributable to the only child in a family being better protected during the period in China. In this study, nearly two-thirds of patients sustaining TSCIs were over the age of 55 years. Similarly, in Japan, the majority of patients sustaining SCIs were over the age of 50 years (52). This is primarily due to early spinal degenerative changes such as stenosis, spondylolisthesis, and degenerative disk disease, specifically ossification of the posterior longitudinal ligament as well as an increased prevalence of congenital stenosis, causing a higher risk of SCI following a traumatic event (5, 50, 53). Degeneration of various components of the vertebra is common in the elderly population and may lead to narrowing of the spinal canal (5, 50, 53). In turn, these degenerative changes place people at a greater risk of suffering SCI following a fall or another traumatic event (5, 50, 53). In this study, fall was the primary cause of TSCI, which, in turn, supports this explanation.

Although the incidence of TSCI in this survey still showed weak male preponderance without statistical significance, the absolute male preponderance in other studies (5–10, 26, 32–39, 41–48) was likely to be weakened by aging and the one-child policy in China. In contrast, the prevalence of SCI in males was 1.9 times that in females, implying same risk of injury and different postinjury survival between sexes in China.

Indeed, from the perspective of injury occupations, farmers and migrant workers from the villages accounted for 61.2% of patients with TSCI injury. It was explained to some extent why incidence of TSCI in rural areas was higher than that in urban areas in China. On the contrary, the prevalence of TSCI in urban areas was slightly higher than that in rural areas. It could reflect the higher healthcare level for TSCI in urban China to a certain extent.

Interestingly, both the incidence and the prevalence of TSCI across western, central, and eastern areas keep a consistent order from high to low with the increasing economy. Likewise, the tetraplegia incidence of traumatic SCI in Taiwan decreases with good economic performance, which may be resulted from the provision of public goods and services, possibly through improvements in the infrastructure of transportation and construction (54).

Data from the previous incidence survey of TSCI in Taiwan showed that the incidence of TSCI increased from 14.6 in 1978–1981 (8) to 150.6 in 1998–2008 (3) in Taiwan. With the increase of aging and motor vehicle, the incidence of TSCI in China will be expected to increase in the future.

### External Causes

Generally, MVCs and falls are the two major causes of TSCI (3, 5–10, 21, 26, 32–48). Although both the MVCs and falls may swap each other in the top two in previous studies, MVCs were the largest cause of SCI in the majority of the previous studies (3, 8–10, 21, 26, 32, 34–37, 39, 43, 45, 46). In previous studies with TSCI in a bimodal distribution of age (10, 34, 37–39, 41, 43), a first peak for young adults was attributable to MVCs, while a second peak in elderly people aged 65 years and older can be mainly ascribed to falls. Consistent with other studies in the mainland of China (5–7), falls in this survey was the primary cause of TSCI, followed by MVCs. On the contrary, the primary cause of TSCI in Taiwan is MVCs, followed by falls (3, 8–10). A study from Tianjin testified that the leading cause of TSCI had shifted from MVCs during the period of 1997–2007 to falls during the period of 2008–2016 with the rapid aging of Chinese society and effectively traffic management. It was also observed that compared with the elderly, young and middle-aged people were more likely to become injured in traffic accidents (12). This shift in external cause of TSCI also contributed to the increase of the mean age at the time of injury. In this study, the mean age at the time of injury increased from 43.7 ± 17.1 years among the 394 prevalent cases to 56.0 ± 17.0 years among the 34 incident cases. In China, firearms are strictly controlled, so such injury was scarce. Compared with developed countries, sport injuries were also uncommon in China because of low prevalence of certain risky sports such as rugby, diving, and motor racing (6). The same findings were found in this survey.

### Strengths and Limitations

To the best of our knowledge, this is the first large-scale sampling survey on epidemiology of patients with TSCI with temporary or permanent sensory deficits, motor deficits, or bladder/bowel dysfunction in population in China including ~600,000 people with better representativeness of the Chinese population. However, it also had many shortcomings. First, it may be difficult to assure the sufficient validity of TSCI epidemiological survey based on the sample calculation of the national cerebrovascular disease epidemiological survey, due to the relatively low incidence of TSCI. Fewer TSCI cases also limit further subgroup analysis. Second, nearly a third of the 394 cases with diagnosis of TSCI were not confirmed with CT/MRI imaging. Third, a recall bias existed in this cross-sectional survey because we could not obtain accurate information of TSCI on dead cases within the defined period of incidence, even though we examined all the deaths in the period. In our survey on incidence of TSCI, only 2 cases of incident TSCI were from deaths from 66 road traffic accidents and 31 falls; therefore, the incidence of TSCI may be underestimated. Finally, this population-based study is based on medical records from hospitals of different grades as well as injury history. Considering the feasibility in population, we could not collect scores at injury of the America Spinal Injury Association Impairment Scale (AIS)/Frankel grade, especially for cases not accessing to hospital. Accordingly, we are unable to differentiate whether a patient is a complete or incomplete SCI.

## Conclusion

In summary, it is estimated that there are 759,302 prevalent patients with TSCI in total and 66,374 new TSCI cases annually in China. Falls and MVCs are still 2 major external causes for TSCI in China. The burden of TSCI in China will be expected to rise with increasing falls in the elderly and increasing use of motor vehicles. These findings may provide a data reference for relevant health administrative departments or professional associations tasked with healthcare policymaking, resources allocation, or disease management in patients with TSCI.

## Data Availability Statement

The original contributions presented in the study are included in the article/Supplementary Material, further inquiries can be directed to the corresponding author.

## Ethics Statement

The studies involving human participants were reviewed and approved by the Ethics Committee of the Beijing Tiantan Hospital affiliated with the Capital Medical University (Ethic ID: KY2013-006-01). Written informed consent to participate in this study was provided by the participants' legal guardian/next of kin.

## Author Contributions

BJ and WW were the principal investigators responsible for the survey, as such, had full access to all the data in the study, and took responsibility for the integrity of the data and the accuracy of the data analysis. BJ performed the statistical analysis and manuscript writing. All authors contributed to the study conception and design, its implementation and field works, data collection and analysis, discussed the findings, and approved the final version for publication.

## Funding

This study was funded by the Ministry of Science and Technology and the Ministry of Health of the People's Republic of China under Grant No. 2011BAI08B01 of the National Key Technology R&D Program and the National Natural Science Foundation of China under Grant No. 81571130090.

## Conflict of Interest

The authors declare that the research was conducted in the absence of any commercial or financial relationships that could be construed as a potential conflict of interest.

## Publisher's Note

All claims expressed in this article are solely those of the authors and do not necessarily represent those of their affiliated organizations, or those of the publisher, the editors and the reviewers. Any product that may be evaluated in this article, or claim that may be made by its manufacturer, is not guaranteed or endorsed by the publisher.
