# Supplementary material for: Prevalence, Incidence, and External Causes of Traumatic Spinal Cord Injury in China: A Nationally Representative Cross-Sectional Survey
Source: Front Neurol. 2022 Jan 20;12:784647. doi: 10.3389/fneur.2021.784647 (PMC8811043; doi:10.3389/fneur.2021.784647)
Supplement: Supplementary file 1 [file Table_1.docx]

**Prevalence, incidence and external causes of traumatic spinal cord injury in China: a nationally representative cross-sectional survey**

Bin Jiang, Dongling Sun, Haixin Sun, Xiaojuan Ru, Hongmei Liu, Siqi Ge, Jie Fu, Wenzhi Wang

Department of Neuroepidemiology, Beijing Neurosurgical Institute, Beijing Tiantan Hospital, Capital Medical University, Beijing, China (Prof B Jiang, MD, D Sun, MD, PhD, H Sun, MD, PhD, X Ru, MD, PhD, H Liu, MD, S Ge, MD, PhD, J Fu, BA, Prof W Wang, MD),

Beijing Municipal Key Laboratory of Clinical Epidemiology, Beijing, China (Prof B Jiang, MD, D Sun, MD, PhD, H Sun, MD, PhD, X Ru, MD, PhD, H Liu, MD, S Ge, MD, PhD, Prof W Wang, MD),

National Office for Cerebrovascular Diseases (CVD) Prevention and Control in China, Beijing, China (H Liu, MD, Prof W Wang, MD)

**Correspondence to:**

Prof Bin Jiang,

Department of Neuroepidemiology,

Beijing Neurosurgical Institute,

Beijing Tiantan Hospital,

Capital Medical University,

Area 2, Building 1, Room 1003

No. 119, South Fourth Ring Road West, Fengtai District

Beijing 100070, P. R. China

E-mail: [bjyjiang@hotmail.com](mailto:bjyjiang@hotmail.com), [bjyjiang@163.com](mailto:bjyjiang@163.com)

**ORCID number:** Bin Jiang, 0000-0001-5808-7178

**Running title:** Spinal cord trauma in China

| **Supplementary Table 1 Demographic characteristics of the confirmed cases of traumatic spinal cord injury (TSCI) and non-TSCI cases from participants with self-reported history of traumatic brain or spinal cord injury** | | | |
| --- | --- | --- | --- |
|  | TSCI | Non TSCI | Total |
| Characteristic | 394 | 2812 | 3206 |
| Age at injury onset (years, mean±SD) | 43.44±17.10 | 40.02±18.88 | 40.43±18.71 |
| Age group at injury onset, n (%) |  |  |  |
| 0~ | 32(8.1%) | 347(12.3%) | 379(11.8%) |
| 15~ | 45(11.4%) | 304(10.8%) | 349(10.9%) |
| 25~ | 64(16.2%) | 443(15.8%) | 507(15.8%) |
| 35~ | 69(17.5%) | 551(19.6%) | 620(19.3%) |
| 45~ | 76(19.3%) | 515(18.3%) | 591(18.4%) |
| 55~ | 68(17.3%) | 379(13.5%) | 447(13.9%) |
| 65~ | 26(6.6%) | 192(6.8%) | 218(6.8%) |
| 75~ | 14(3.6%) | 81(2.9%) | 95(3.0%) |
| Sex |  |  |  |
| Sex, n (%) |  |  |  |
| Men | 256(65.0%) | 1805(64.2%) | 2061(64.3%) |
| Women | 138(35.0%) | 1007(35.8%) | 1145(35.7%) |
| Education, n (%) |  |  |  |
| Primary school or preschool | 199(50.5%) | 1534(54.6%) | 1733(54.1%) |
| Middle school | 182(46.2%) | 1155(41.1%) | 1337(41.7%) |
| College and higher | 13(3.3%) | 119(4.2%) | 132(4.1%) |
| Unknown | 0 | 4(0.1%) | 4(0.1%) |
| Occupation, n (%) |  |  |  |
| Students | 3(0.8%) | 84(3.0%) | 87(2.7%) |
| Worker | 18(4.6%) | 145(5.2%) | 163(5.1%) |
| Farmer or farmer worker | 241(61.2%) | 1739(61.8%) | 1980(61.8%) |
| Employee | 14(3.6%) | 120(4.3%) | 134(4.2%) |
| Self-employed | 39(9.9%) | 155(5.5%) | 194(6.1%) |
| Retiree or homemaker | 77(19.5%) | 524(18.6%) | 601(18.7%) |
| Other or unknown | 2(0.5%) | 45(1.6%) | 47(1.5%) |
| Place of residence, n (%) |  |  |  |
| Urban | 197(50.0%) | 1307(46.5%) | 1504(46.9%) |
| Rural | 197(50.0%) | 1505(53.5%) | 1702(53.1%) |
| Geographic Location, n (%) |  |  |  |
| Eastern China | 88(22.3%) | 617(21.9%) | 705(22.0%) |
| Central China | 177(44.9%) | 1096(39.0%) | 1273(39.7%) |
| Western China | 129(32.7%) | 1099(39.1%) | 1228(38.3) |
