# Supplementary material for: Prevalence, Incidence, and External Causes of Traumatic Spinal Cord Injury in China: A Nationally Representative Cross-Sectional Survey
Source: Front Neurol. 2022 Jan 20;12:784647. doi: 10.3389/fneur.2021.784647 (PMC8811043; doi:10.3389/fneur.2021.784647)
Supplement: Supplementary file 2 [file Table_2.docx]

**Prevalence, incidence and external causes of traumatic spinal cord injury in China: a nationally representative cross-sectional survey**

Bin Jiang, Dongling Sun, Haixin Sun, Xiaojuan Ru, Hongmei Liu, Siqi Ge, Jie Fu, Wenzhi Wang

Department of Neuroepidemiology, Beijing Neurosurgical Institute, Beijing Tiantan Hospital, Capital Medical University, Beijing, China (Prof B Jiang, MD, D Sun, MD, PhD, H Sun, MD, PhD, X Ru, MD, PhD, H Liu, MD, S Ge, MD, PhD, J Fu, BA, Prof W Wang, MD),

Beijing Municipal Key Laboratory of Clinical Epidemiology, Beijing, China (Prof B Jiang, MD, D Sun, MD, PhD, H Sun, MD, PhD, X Ru, MD, PhD, H Liu, MD, S Ge, MD, PhD, Prof W Wang, MD),

National Office for Cerebrovascular Diseases (CVD) Prevention and Control in China, Beijing, China (H Liu, MD, Prof W Wang, MD)

**Correspondence to:**

Prof Bin Jiang,

Department of Neuroepidemiology,

Beijing Neurosurgical Institute,

Beijing Tiantan Hospital,

Capital Medical University,

Area 2, Building 1, Room 1003

No. 119, South Fourth Ring Road West, Fengtai District

Beijing 100070, P. R. China

E-mail: [bjyjiang@hotmail.com](mailto:bjyjiang@hotmail.com), [bjyjiang@163.com](mailto:bjyjiang@163.com)

**ORCID number:** Bin Jiang, 0000-0001-5808-7178

**Running title:** Spinal cord trauma in China

| **Supplementary Table 2 Prevalence of traumatic spinal cord injury (TSCI) from different regions or countries** | | | | | | |
| --- | --- | --- | --- | --- | --- | --- |
| Author, date, reference | Region/Country | Design | TSCI definition | Population | Point prevalence date /period | Prevalence (1/1000000) |
| Rahimi-Movaghar et al, 2009[21] | Tehran, Iran | Cross-sectional survey, point prevalence | Not defined | 9,006 persons from 2425 families | September 1, 2007 | 440 per million population |
| Razdan et al,1994[22] | Kashmir, India | Cross-sectional survey, point prevalence | Not defined | The rural population of 63,645 living in the mountainous Kuthar Valley of south Kashmir, northwestern India | November 1, 1986 | 236.0 per million population |
| Minaire et al,1978[23] | Rhone-Alpes Region, France | Calculated by multiplying incidence by average life duration (Henry Gabrielle Hospital). | Not defined | - | - | 250 per million population |
| Dahlberg et al,2005[24] | Helsinki, Finland | Cross-sectional survey, point prevalence | ICD-9 codes 806, 952, and 9072A until 1995; ICD-10 codes S14.0-2, S24.0-2,S34.0-3, and T91.3 after 1995 | 546 000 inhabitants in Helsinki | January 1, 1999 | 280 per million |
| Hagen et al,2010[25] | Western Norway | Cross-sectional survey, point prevalence | Acute, traumatic lesion of the spinal cord resulting in motor and/or sensory deficit and/or bowel/bladder dysfunction, either temporary or permanent. | 545 533 inhabitants on 1 January 2002 | January 1, 2002 | 365 per million population |
| Knútsdóttir et al,2012[26] | Iceland | A retrospective review of hospital records on all admissions due to SCIs, point prevalence | ICD-9 codes 806 and 952, and IiCD-10 codes S14, S24, and S34 since 1997. Patients with isolated injuries of the nerve roots and patients with symptoms lasting for 2 weeks were excluded | the Icelandic population | December 31,2009 | 526 per million population |
| O'Connor et al,2005[27] | Australia | Calculated by multiplying current incidence by disease duration (Australian Spinal Cord Injury Register). | Not defined | - | 1997 | 681 per million population |
| New et al,2015[28] | Australia | Population modeling using cohort survival | Not defined | - | June 30, 2011 | 490-886 per million population |
| DeVivo et al,1980[29] | United States of America | Calculated by multiplying annual incidence with life duration. | Not defined | - | - | 906 per million population |
| Harvey et al,1990[30] | United States of America | Mixed-mode sampling design was used to survey both non-institutionalized and institutionalized populations. Non-institutionalized: area segments were selected with primary sampling health facilities within primary sampling units. Institutionalized: nursing and long-term care were sampled. | Not defined | - | 1988 | 721 per million population |
| Noonan et al,2012[31] | Canada | 2010 Canadian discharge incidence rates on historical demographics using a cohort survival model and age-specific mortality rates. | Not defined | - | 2010 | 1298 per million population |
| Present survey | China | Cross-sectional survey, point prevalence | Acute, traumatic lesion of the spinal cord resulting in motor and/or sensory deficit and/or bowel/bladder dysfunction, either temporary or permanent. | 596536 persons | August 31, 2013 | 569.7 per million population (95% CI: 514.2-630.4) |
