# Supplementary material for: Prevalence, Incidence, and External Causes of Traumatic Spinal Cord Injury in China: A Nationally Representative Cross-Sectional Survey
Source: Front Neurol. 2022 Jan 20;12:784647. doi: 10.3389/fneur.2021.784647 (PMC8811043; doi:10.3389/fneur.2021.784647)
Supplement: Supplementary file 3 [file Table_3.docx]

**Prevalence, incidence and external causes of traumatic spinal cord injury in China: a nationally representative cross-sectional survey**

Bin Jiang, Dongling Sun, Haixin Sun, Xiaojuan Ru, Hongmei Liu, Siqi Ge, Jie Fu, Wenzhi Wang

Department of Neuroepidemiology, Beijing Neurosurgical Institute, Beijing Tiantan Hospital, Capital Medical University, Beijing, China (Prof B Jiang, MD, D Sun, MD, PhD, H Sun, MD, PhD, X Ru, MD, PhD, H Liu, MD, S Ge, MD, PhD, J Fu, BA, Prof W Wang, MD),

Beijing Municipal Key Laboratory of Clinical Epidemiology, Beijing, China (Prof B Jiang, MD, D Sun, MD, PhD, H Sun, MD, PhD, X Ru, MD, PhD, H Liu, MD, S Ge, MD, PhD, Prof W Wang, MD),

National Office for Cerebrovascular Diseases (CVD) Prevention and Control in China, Beijing, China (H Liu, MD, Prof W Wang, MD)

**Correspondence to:**

Prof Bin Jiang,

Department of Neuroepidemiology,

Beijing Neurosurgical Institute,

Beijing Tiantan Hospital,

Capital Medical University,

Area 2, Building 1, Room 1003

No. 119, South Fourth Ring Road West, Fengtai District

Beijing 100070, P. R. China

E-mail: [bjyjiang@hotmail.com](mailto:bjyjiang@hotmail.com), [bjyjiang@163.com](mailto:bjyjiang@163.com)

**ORCID number:** Bin Jiang, 0000-0001-5808-7178

**Running title:** Spinal cord trauma in China

| **Supplementary Table 3 Incidence of traumatic spinal cord injury (TSCI), etiology and demographic characteristics of TSCI patients from different regions or countries** | | | | | | | | |
| --- | --- | --- | --- | --- | --- | --- | --- | --- |
| Author, date, reference | Region/Country | Period | Design/methods | Incidence (95% CI; per million population per year) | Leading causes (%) | Second causes (%) | Mean/median age | Male-to-female ratio |
| Selassie et al, 2015[32] | South Carolina, USA | 1998–2012 | A Population-based Study of TSCI surveillance and follow-up registry in persons older than 21 years | 70.8 (average) during 1993-2012; 66.9 in 1998; 111.7 in 2012. | MVCs: 35.4% on average | Falls: 32.2% on average | 51.5 | 2.88:1 |
| Jain et al, 2015[33] | USA | 1993–2012 | Survey data from the US Nationwide Inpatient Sample databases in persons older than 15 years | 54.0 (53.0-54.0) in 2012 | Falls: 40.4% in 2012 | MVCs: 31.0% in 2012 | 50.5 | 2.41:1 |
| Dryden et al, 2003[34] | Alberta, Canada | 1997–2000 | Administrative data from the Alberta Ministry of Health and Wellness, records from the Alberta Trauma Registry, and death certificates from the Office of the Medical Examiner. | 52.5 (47.7-57.4) | MVCs: 56.4% | Falls: 19.1% | 35.0 (median) | 2.52:1 |
| Pickett et al, 2006[35] | Canada | 1997–2001 | Retrospectively reviews on hospital records of all patients with traumatic SCI between January 1997 and June 2001 among a catchment population of 924,257 people. | 40.9 (average) | MVCs: 35.1% | Falls: 31.1% | 42.2 | 2.88:1 |
| Lenehan et al, 2012[36] | British Columbia, Canada | 1995-2004 | Retrospective observational study utilizing prospectively collected population-based data among a population of approximately 4.2 million. | 35.7 (average) | MVCs: 51.4% | Falls: 28.5% | 35.0 (median) | 4.40:1 |
| Thompson et al. 2015[37] | Québec, Canada | 2000–2011 | Data concerning T-SCI patients was retrieved from the Québec Trauma Registry in this retrospective cohort study. | 16.9 (average) | MVCs: 41.5% | Falls: 35.0% | 46.2 | 3.95:1 |
| Montoto-Marqués et al, 2017[38] | Galicia, Spain | 1995–2014 | Observational study with prospective and retrospective monitoring. | 21.7 (average) | Falls: 54.2% | MVCs: 37.0% | 50.2 | 3.24:1 |
| O'Connor, 2002[39] | Australia | 1998–1999 | Australian SCI register | 14.5 | MVCs: 42.6% | Falls: 31.3% | - | 3.17:1 |
| Majdan et al, 2016[40] | Austria | 2002–2012 | TSCI-related deaths and hospital admissions in Austria 2002–2012 were obtained from Statistics Austria. | 17.0 | Falls: 49.0% | Injuries at home: 40.0% | - | 1.86 |
| Chamberlain et al, 2015[41] | Switzerland | 2005–2012 | Data from a retrospective study of medical files from all four specialized rehabilitation centers for SCI in Switzerland. | 18.0 | Falls: 39.1% | Sports & leisure: 26.5% | 48.0 | 2.90:1 |
| Nijendijk et al, 2014[42] | Netherlands | 2010 | Data from a retrospective study of the Dutch National acute-care hospital database. | 14.0 | Falls: 53.0% | MVCs: 21.6% | 56.2 | 2.85:1 |
| Knútsdóttir et al, 2012[26] | Iceland | 1975–2009 | Retrospective population-based epidemiological study. | 30.0 (average) in 1975-1979; 12.5 (average) in 1995-1999; 33.5 (average) in 2005-2009 | MVCs: 42.5% | Falls: 30.9% | 38.0 | 2.57:1 |
| O'Connor and Murray, 2006[43] | Ireland | 2000 | Prospective data collection on all patients with spinal cord injury (SCI) admitted for a comprehensive management programme. | 13.1 | MVCs: 50.0% | Falls: 37.0% | 37.0 (median) | 6.69:1 |
| Sabre et al, 2012[44] | Estonia | 1997–2007 | Retrospective population-based cohort study. | 39.7 (36.6–43.0) | Falls: 41.0% | MVCs: 29.4% | 39.0 | 5.47:1 |
| Sabre et al, 2015[45] | Estonia | 2005–2007 | Medical records of patients with TSCI from all hospitals in Estonia from 2005 to 2007 and the data of the victims of TSCI who died before hospitalization. | 97.0 (87.4–106.6) | MVCs: 53.3% | Falls: 27.4% | 44.4 | 3.89:1 |
| Löfvenmark et al, 2015[46} | Botswana | 2011–2013 | Descriptive study with a cross-sectional design. | 13.0 | MVCs: 68.0% | Violence: 16.0% | - | 2.45:1 |
| Taşoğlu et al, 2018[47] | Turkey | 2013–2014 | Retrospective study. | 8.1–21.3 | Falls: 34.4& | MVCs: 30.2% | 38.3 | 2.31:1 |
| Rahimi-Movaghar et al, 2009[21] | Tehran, Iran | 2003–2008 | A population-based study | 44.0 | MVCs: 75.0% | Falls: 25.0% | 31.0 | 1.00:1 |
| Katoh et al, 2014[48] | Japan | 2011–2012 | Retrospective questionnaire-based epidemiological study. | 121.4 in 2011; 117.1 in 2012 | Falls: 62.1% in 2011; 54.9& in 2012 | MVCs: 23.2% in 2011; 27.5% in 2012 | 67.6 in 2011; 64.3 in 2012 | 2.65 in 2011; 2.64:1 in 2012 |
| Ning et al, 2011[6] | Tianjin, People's Republic of China | 2004–2008 | A retrospective epidemiological study. All TSCI patients aged 15 years or older who were admitted to tertiary hospitals in Tianjin from 2004 to 2008. | 23.7 | Falls: 56.9% | MVCs: 34.1% | 46.0 | 5.63:1 |
| Li et al, 2011[5] | Beijing, People's Republic of China | 2002 | A retrospective epidemiological study. Data of all ATSCI patients who were injured in Beijing in 2002 from sample hospitals were collected for detailed investigation. | 60.6 | Falls: 45.8% | MVCs: 26.9% | 41.0 | 3.13:1 |
| Du et al, 2020[7] | Xi'an, People's Republic of China | 2014-2018 | A hospital-based retrospective epidemiological study. The medical records of the all spine centers or orthopedic centers in Xi’an were collected according to the International Classification of Disease Version 10 (ICD-10) and diagnostic code of TSCI. | 41.0 (36.6–45.3) | Falls: 62.2% | MVCs: 26.6% | 50.1 | 2.90:1 |
| Chen et al, 1985[8] | Taipei, Taiwan, People's Republic of China | 1978-1981 | All record from general hospitals in Taipei. Included all spinal cord lesions. | 14.6 | MVCs: 44.5% | Falls: 28.5% | 36.2 | 4.9:1 |
| Lan et al, 1993[9] | Hualien county, Taiwan, People's Republic of China | 1986-1990 | Traumatic SCI in 4 hospitals in Hualien county. Exclusion Criteria: People who died before hospitalization; non-traumatic SCI; patients with transient paralysis; non-residents of Hualien. | 56.1 | MVCs: 61.6% | Falls: 23.3% | male: 44.0; female: 46.0 | 4:1 |
| Chen et al, 1997[10] | Taiwan, People's Republic of China | 1992-1996 | Admissions in one the 113 hospitals (including 11 medical centers, 50 regional general hospitals, 52 local general hospitals) | 18.8/million/year (1992-1996); 24.5/million/year (1993); 19.6/million/year (1994); 18.2/million/year (1995); 17.2/million/year (1996) | MVCs: 46.0% | Falls: 44.1% | 46.1 | 3:1 |
| Wu et al, 2012 [3] | Taiwan, People's Republic of Chin | 1998-2008 | SCI patients at least 20 years of age in the National Health Insurance Research Database of Taiwan. | 150.6/million/year | MVCs: 58.8% | Falls: 34.8% | -- | 1.52:1 |
| Present survey | People's Republic of China | 2012.9.1-2013.8.31 | A retrospective population-based epidemiological study. | 49.8 (34.4-70.7) | Falls: 55.2% | MVCs: 26.5% | 43.7 | 1.86:1 |
