# Supplementary material for: Prevalence, Incidence, and External Causes of Traumatic Spinal Cord Injury in China: A Nationally Representative Cross-Sectional Survey
Source: Front Neurol. 2022 Jan 20;12:784647. doi: 10.3389/fneur.2021.784647 (PMC8811043; doi:10.3389/fneur.2021.784647)
Supplement: Supplementary file 4 [file Data_Sheet_1.PDF]

Questionnaire for the National Epidemiological Survey on Traumatic Brian and Spinal Cord Injuries

(Completed by CDC investigators)

Name of survey site (district/county):_______________	Code of survey site: □□□□□□
Name of township/sub-district:____________________	Code of township/sub-district: □
Name of village/residential community:_____________	Code of village/residential community: □
Name of family contact person:____________________	Family code: □□□□
Telephone 1 for family contact person:______________	Telephone 2 for family contact person:_______________
The investigator must complete the above information before completing the “Registration Form for Family Members”. The codes of the survey sites are uniformly assigned by the national project team (Appendix 1). The codes of the townships/sub-districts and villages/residential communities are assigned by the responsible person at the local CDC and will be distributed to investigators before the survey begins.	
Signature of the investigator:______________________<br>Date:	Signature of QC staff at survey site:<br>Date:
Signature of provincial CDC/department of neurology/national project team_________________________<br>Date:	
Investigator and quality control staff should sign their names after completing or checking the Preliminary Screening Form.	

I Registration of family members

Information for one permanent family member should be completed on each line. The information for the head of the household should be filled out first, followed by other members from older to younger.

Code of family members	Name	Sex (1=male, 2=female)	Date of birth	Relationship to the head of household	Local hukou (1=Yes, 2=No)	Living in the current place of residence for ≥6 months (1=Yes, 2=No)
**1**						
**2**						
**3**						
**4**						
**5**						
**6**						

Notes:
Codes for the relationship to the head of the household: 1=household head; 2=spouse; 3=child; 4=child's spouse; 5=grandson/granddaughter; 6=parent; 7=parent-in-law; 8=brother/sister; 9=grandfather/grandmother; 10=other relative; 11=Not related (friend, nanny, etc.)

If the number of family members exceeds six, a second Preliminary Screening Form should be used. The basic information should be completed and the serial number for family members should be changed accordingly. For example, the number "1" should be marked off, and "7" recorded nearby; similarly, mark off "2" and add "8" and so on.

No.	Items	Code of family member					
		1	2	3	4	5	6
**II Family members' basic information**							
1	Interview mode:<br>1=Face-to-face interview; 2=Telephone;<br>3=Answered by a family member on behalf (indicate the relationship)						
	Record the corresponding code in the space provided [1=Face-to-face interview; 2=Telephone; 3=Answered by a family member on behalf]; if the code is 3, indicate the relationship (e.g., father, mother, son, daughter, grandson, etc.)						
2	Ethnic groups:<br>1=Han; 2=Zhuang; 3=Manchu;<br>4=Hui; 5=Miao; 6=Uighur;<br>7=Yi; 8=Tujia; 9=Mongolian;<br>10=Korean; 11=Tibetan;<br>88=Other ethnic group (please specify)						
	Record the corresponding number in the space provided. If the participant does not belong to any of the listed ethnic groups, write "88" and record the specific ethnic group.						
3	Education background:<br>1=Preschool; 2=Not attended school<br>3=Not graduated from primary school;<br>4=Graduated from primary school;<br>5=Graduated from middle school;<br>6=Graduated from high school/technical school/secondary technical school;<br>7=Graduated from junior college;<br>8=Graduated from college;<br>9=Graduated with a master's/doctoral degree						
	Education background refers to the highest education level or the academic qualifications matching the existing degree of literacy. For a participant who has not graduated, record the education level they completed (e.g., for a grade 1 student in high school, select "5=Graduated from middle school."<br><br>1 Preschool: Infants or children not yet old enough to attend school.<br>2 Not attended school: Individuals who have never attended a school.<br>3 Not graduated from primary school: Individuals who attended primary school but did not graduate. This applies to those who have achieved basic literacy standards (e.g., able to read popular magazines or write notes).<br>4 Graduated from primary school: Individuals who graduated from primary school but did not attend middle school, attended middle school but did not graduate, or students still studying in middle school.<br>5 Graduated from middle school: Individuals who graduated from middle school but did not attend high school, attended a high school but did not graduate, or students still studying in high school. Technical school is equivalent to middle school.<br>6 Graduated from high school/technical school/secondary technical school: Individuals who graduated from high school (general high school/technical school/secondary technical school), who studied at college but did not graduate, or students still studying at college (including junior college).<br>7 Graduated from junior college: Individuals who graduated from a state licensed junior college.<br>8 Graduated from college: Individuals who graduated from a state licensed college. This applies to individuals who received college education via self-teaching, evening college, TV universities, correspondence college, and other institutions entitled to grant a college degree. It also applies to individuals who attended a graduate program but failed to obtain a degree and current master's/doctoral candidates.						

	9 Graduated with a master's/doctoral degree: Individuals who received a graduate education and earned a master's/doctoral title.						
4	Marital status:<br>1=Unmarried: 2=Married; 3=Divorced;<br>4=Widowed; 5=Not applicable; 88=Other						
	1 Unmarried: Individuals who have never been married.<br>2 Married: Individuals who have been married and are living with their spouses.<br>3 Divorced: Individuals who are divorced and have not re-married.<br>4 Widowed: Individuals who lost their spouses and have not re-married.<br>5 Not applicable: Minors.<br>88 Other: Individuals with unknown marital status and those refuse to answer.						
5	Current occupation:<br>1=Worker; 2=Farmer; 3=Student;<br>4=Civil servant; 5=Professional;<br>6=Staff or manager of an enterprise or institution; 7=Active military personnel;<br>8=Self-employed; 9=Freelancer;<br>10=Retired; 11=Housework; 12=Unemployed;<br>13=Preschool children; 88=Other (please specify:)						
	Job-holders and non-job-holders are included. For job-holders with several different occupations (part-time jobs), the occupation with a fixed working time and that is the major income source is regarded as the participant's occupation. Individuals who took a job after retirement may also be regarded as a job-holder if the duration of the job exceeds one year.<br>Job-holders:<br>1 Worker: Staff and other personnel engaged in the operation of production/transport equipment, mineral exploration, mining, manufacturing, construction, and transportation equipment operation.<br>2 Farmer: Individuals involved in the production, management, and product processing in fields including agriculture, forestry, animal husbandry, fishery, and the water conservancy industry.<br>3 Students: Individuals who are studying at primary school, middle school, high school, or college (including graduate students).<br>4 Civil servants: Heads of government agencies, mass organizations, enterprises and institutions; leaders (involved in decision-making and management) in the Communist Party of China Central Committee and local party organizations at all levels, standing committees of the people's congresses, people's political consultative conferences, people's courts, people's procuratorates, government agencies, democratic parties, trade unions, the Communist Youth League, women's federations, community organizations and their working bodies, enterprises, and institutions.<br>5 Professionals and technical personnel: Personnel engaged in scientific research and professional and technical work, including researchers, science and technology management and support personnel, aircraft and ship technicians, medical staff, legal staff, economic management professionals, teachers, teaching assistants, artists, and sports staff.<br>6 Government employees and enterprise staff: Individuals responsible for administrative affairs in government agencies, mass organizations, enterprises and institutions; or engaged in security, fire protection, telecommunications, and other relevant work.<br>7 Active military personnel: Military personnel currently serving in the People's Liberation Army or the armed police forces.<br>8 Self-employed: Individuals engaged in commerce, catering, tourism and entertainment, transport, auxiliary medical services, and other service work.<br>9 Freelancer: Individuals without regular employment.<br>10 Retired: Individuals who have retired from their positions according to government policies and currently have no regular job. Individuals who have been employed for more than 1 year after retirement shall be registered as job-holders using their current occupation.<br>11 Housework: Individuals (e.g., housewives and unemployed persons) who are mainly engaged in household activities such as cooking and laundry.<br>12 Unemployed: Jobless individuals and students who have graduated from a school but have not yet						

	found a job. Retired individuals are not included.<br>13 Preschool children<br>88 Other: Individuals who cannot be classified into the above categories.							
6	Health care payment modes (multiple selection):<br>1=Basic medical insurance for urban workers;<br>2=Basic medical insurance for urban residents;<br>3=Commercial medical insurance;<br>4=New rural cooperative medical system;<br>5=Publicly funded health care system;<br>6=Out-of-pocket payment;<br>88=Other social insurance; 99=Unknown							
	The investigator should read out the answer options, and judge whether the participant's answer is correct based on his or her occupation.<br>1 Basic medical insurance for urban workers: All urban employers and employees (typically urban residents with regular jobs) must participate in basic medical insurance.<br>2 Basic medical insurance for urban residents: A medical insurance system for minors not covered by the basic medical insurance for urban workers, and for unemployed residents.<br>3 Commercial medical insurance: Includes various commercial types of insurance.<br>4 New rural cooperative medical system: Mutually beneficial health care funding system for rural residents. Organized, guided, and supported by the government, with farmers as voluntary participants; jointly funded by farmers, villages, and governments.<br>5 Publicly funded health care system: Social security system provided by the government in an attempt to secure formal government employees. The costs of health care and prevention services are paid by the national financial system. Currently this system is relatively rare.<br>6 Out-of-pocket payment: The individual pays their medical costs by themselves.<br>88 Other: Such as major disease relief mechanism. 99 Unknown.							
**III History of brain/spinal cord trauma**								
24	Have you ever suffered from relatively severe brain/spinal cord trauma (concussion and more severe conditions)?<br>1=Yes; 2=No (Skip to VI Death of family members);<br>99=Unknown (Skip to VI Death of family members)							
	Aim: To determine previous history of brain/spinal cord trauma; mainly refers to relatively severe brain/spinal cord trauma.<br>1 Yes: The participant has suffered from relatively severe brain/spinal cord trauma.<br>2 No: The participant has not experienced relatively severe brain/spinal cord trauma. If this answer is selected, skip to VI Death of family members.<br>99 Unknown: The participant is not sure whether he/she has suffered from relatively severe brain/spinal cord trauma. If this answer is selected, skip to VI Death of family members.							
25	Times and dates of severe brain/spinal cord trauma:	1st time						
		2nd time						
	Aim: To determine the times and timing of severe brain/spinal cord trauma. If severe brain/spinal cord trauma was suffered more than once, record the date (e.g., 2013/01/01) of these experiences. If severe brain/spinal cord trauma was suffered more than three times, record the most recent two times.							
26	Site of severe brain/spinal cord trauma:	1st time						

	1=Brain; 2=Spinal cord;<br>3=Both brain and spinal cord;<br>99=Unknown	2nd time						
	Aim: To determine the site of severe brain/spinal cord trauma.							
27	27 If you have suffered from severe brain/spinal cord trauma, which symptoms did you experience? [YOU MAY CHOOSE MORE THAN ONE RESPONSE]<br>1=Unconsciousness or coma;<br>2=Cannot recall the cause or conditions of trauma after consciousness was recovered;<br>3=Paralysis or weakness of limbs or trunk;<br>4=Sensory disturbance of the limbs or trunk;<br>5=Fecal/urinary incontinence or retention;<br>6=The above conditions were absent;<br>99=Unclear	1st time						
		2nd time						
	Aim: To determine symptoms following severe brain/spinal cord trauma.<br>1 Unconsciousness or coma: There was no communication with the outside world.<br>2 Cannot recall the cause or conditions of trauma after consciousness was recovered: refers to retrograde amnesia (i.e., the patient cannot recall the cause or conditions of the trauma after transient unconsciousness);<br>3 Paralysis or weakness of limbs or trunk: Symptoms may have occurred on one or both sides;<br>4 Sensory disturbance of limbs or trunk: Symptoms may have occurred on one or both sides;<br>5 Fecal/urinary incontinence or retention: The participant may have suffered from fecal/urinary incontinence or retention and experienced difficulty defecating and/or urinating.<br>99 Unclear: The participant does not know whether such symptoms existed.							
28	Cause of brain/spinal cord trauma:<br>1=Road traffic accident; 2=Fall;<br>3=Strike 4=Gun shot<br>5=Bombing 99=Unclear;<br>88=Other (please specify:)	1st time						
		2nd time						
	Aim: To determine the causes of brain/spinal cord trauma.<br>1 Road traffic accident: Collision or crash that occurred on the road, involving at least one moving vehicle and leading to fatal or non-fatal injuries.<br>2 Fall: Slipping, tripping, or falling on the same level (e.g., slipping due to ice on road), or falling from one level to another (e.g., falling from a considerable height).<br>3 Striking: Mainly refers to being hit with a hard object (e.g., hit or kicked with fists, elbows, or feet).<br>4 Gun shot: Injuries caused by a gun shot.<br>5 Bombing: Injuries caused by bombing.<br>88 Other: Injuries that cannot be classified into the above types (please specify).<br>99 Unclear: The participant does not know the exact cause of brain/spinal cord trauma.							
29	Management after brain/spinal cord trauma:<br>1=Did not go to hospital;	1st time						

	2=Visited a hospital but did not receive CT/MRI;<br>3=Visited a hospital and received CT/MRI, but was not hospitalized;<br>4=Visited a hospital, received CT/MRI and was hospitalized;<br>99=Unknown	2nd time						
	Aim: To determine management following severe brain/spinal cord trauma.<br>1=Did not go to hospital: The participant did not visit a hospital following the brain/spinal cord trauma;<br>2=Visited a hospital but did not receive CT/MRI: The participant visited a hospital following the brain/spinal cord trauma but did not receive a CT/MRI;<br>3=Visited a hospital and received CT/MRI, but was not hospitalized: The participant visited a hospital following the brain/spinal cord trauma, and received a CT/MRI following the brain/spinal cord trauma, but was not hospitalized;<br>4=Visited a hospital, received CT/MRI, and was hospitalized: The participant visited a hospital following the brain/spinal cord trauma, received a CT/MRI, and was hospitalized.							
30	The final diagnosis made by the hospital following the brain/spinal cord trauma: [YOU MAY CHOOSE MORE THAN ONE RESPONSE]<br>1=Concussion;<br>2=Cerebral contusion and laceration;<br>3=Traumatic intracranial hematoma;<br>4= Brain stem injury;<br>5=Spinal cord injury;<br>88= Other (Please specify:);<br>99= Unknown diagnosis	1st time						
		2nd time						
	Aim: To determine the specific diagnosis following the brain/spinal cord trauma. If the participant can provide medical records or diagnosis documentation, that diagnosis should be recorded; if the medical records or diagnosis documentation cannot be provided, the diagnosis should be based on the statement of the participant.<br>1 Concussion: Transient brain dysfunction immediately after the head being hit by an external force. Clinically manifests as transient coma, recent amnesia (retrograde amnesia), and symptoms including headache, nausea, or vomiting. No positive sign may be found during a neurologic examination.<br>2 Cerebral contusion and laceration: Structural damage of brain tissues after head trauma. The injured brain tissue may show spotty or patchy bleeding, rupture, edema or necrosis, often accompanied with focal cerebral edema or diffuse brain swelling at the adjacent sites as well as varying degrees of intracranial hematoma.<br>3 Intracranial hematoma: The most severe type of secondary lesion following brain injury. After brain injury, the blood may accumulate in some parts of the cranial cavity and cause an increase of intracranial pressure when it reaches a considerable volume, leading to a series of clinical symptoms. Based on the location, intracranial hematomas can be classified as epidural hematoma, subdural hematoma, and intracerebral hematoma.<br>4 Brain stem injury: Primary or secondary brain stem injury following traumatic brain injury. Clinically the patient often falls into a coma.<br>5 Spinal cord injury: The majority of spinal cord injuries originate from traffic crashes, falls, violence, or sports-related injuries. The severity of a spinal cord injury is determined by the degree of primary injury after the instantaneous force exerted on the spinal cord, which may cause spinal shock, non-complete injury, complete injury, and spinal cord transection. Common clinical manifestations of spinal cord injury include motor and sensory impairments below the level of injured spinal cord.<br>88 Other: Conditions not included in the above diagnoses (please specify).<br>99 Unclear: The participant does not know their diagnosis of brain/spinal cord trauma.							

VI Death of family members

Have any of your family members passed away since January 1, 2012? ☐ 1= Yes (Continue to complete the following information); 2= No (End)

Code of deceased family member	Name	Sex (1=male; 2=female)	Date of birth	Date of death	Relationship to the head of household	Cause of death/ diagnosis	Name of the hospital where the patient died (If he/she died in another place such as home, work, or farm, please specify)	Level of the hospital where the diagnosis was made	Did he/she have a brain/spinal cord injury before death	Did he/she die of a brain/spinal cord injury
1										
2										
3										
4										

Notes: Codes of the relationship to the head of the household**:** 1=Head of the household; 2=Spouse; 3=Son or daughter; 4=Daughter- or son-in-law; 5=Grandson/granddaughter; 6=Parent; 7=Parent-in-law; 8=Brother or sister; 9=Grandparent; 10=Other relative; 11=Not related (friend, etc.).

Level of the hospital where the diagnosis was made: 1=Community hospital/township health care center/institution clinic; 2=District/county hospital; 3=Provincial/municipal hospital or higher; 4=Not applicable (i.e., not diagnosed in any hospital); 99=Unknown.

Did he/she have a brain/spinal cord injury before death: 1=Yes; 2=No; 99=Unknown.

Did he/she die of a brain/spinal cord injury: 1=Yes; 2=No; 99=Unknown.

Case Review Form

(Completed by a neurologist)

Name of survey site (district/county):____________________	Code of survey site: □□□□□□
Name of township/sub-district:__________________________	Code of township/sub-district: □
Name of village/residential community:___________________	Code of village/residential community: □
Code of household (same as in the preliminary screening questionnaire): □□□□	Code of household member (same as in the preliminary screening questionnaire: □
Name of the reviewed participant:_______________________	Tel:____
ID card number: □□□□□□□□□□□□□□□□□□	
The doctor has told me the purpose and significance of this survey and I voluntarily consent to participate in this survey. Signature:_____________________________________	
The neurologist should complete the above information before completing the form	
Face-to-face interview: □ 1=Yes 2=No (Please describe the reason: ______________________)	
Signature of neurologist:____________________<br>Date:	Signature of QC staff:___________________<br>Date:
Signature of provincial neurologist/national project team QC staff:______________________<br>Date:	

I Brief disease history

If the participant has different diseases, record the brief history of traumatic brain or spinal cord injury only; if there were two or more episodes of traumatic brain or spinal cord injury, record the brief history of the episode with the most recent onset

**1 Description of disease history** (brief description, including time of onset, predisposing factors, status during disease onset, disease progression, and results of special examinations). If possible, record the name of the hospital or review the CT/MRI films or diagnosis reports:<br>____<br>____<br>____<br>____					
**1.1 Date of onset:**___________________________________					□□□□.□□.□□
1.2 Cause of brain/spinal cord trauma:<br>1=Road traffic accident; 2=Fall; 3=Strike 4=Gun shot<br>5=Bombing 99=Unclear;<br>88=Other (please specify:_________________________)					└───┘
Aim: To determine the causes of brain/spinal cord trauma.<br>1 Road traffic accident: Collision or crash that occurred on the road, involving at least one moving vehicle and leading to fatal or non-fatal injuries.<br>2 Fall: Slipping, tripping, or falling on the same level (e.g., slipping due to ice on road), or falling from one level to another (e.g., falling from a considerable height).<br>3 Striking: Mainly refers to being hit with a hard object (e.g., hit or kicked with fists, elbows, or feet).<br>4 Gun shot: Injuries caused by a gun shot.<br>5 Bombing: Injuries caused by bombing.<br>88 Other: Injuries that cannot be classified into the above types (please specify).<br>99 Unclear: The participant does not know the exact cause of brain/spinal cord trauma.					
**2 Sudden neurological symptoms** (1=Yes 2=No 99=Unknown)					
2.1	Unconsciousness or coma	└───┘	2.2	Cannot recall the cause or conditions of trauma after consciousness is recovered	└───┘
2.3	Paralysis or weakness of limbs or trunk	└───┘	2.4	Sensory disturbance of limbs or trunk	└───┘
2.5	Fecal/urinary incontinence or retention	└───┘			
**3 Special examinations** (1=Yes 2=No 99=Unknown)					
3.1	CT/CTA	└───┘	3.2	MRI/MRA	└───┘

II Diagnosis information

**Diagnosis**:<br>1= Concussion 2=Cerebral contusion and laceration 3= Intracranial hematoma 4= Brain stem injury 5= Spinal cord injury	└──┘└──┘
**Location of injury:** 1=Brain; 2=Spinal cord; 3= Both brain and spinal cord.	└──┘

The neurologist should make a diagnosis according to the definitions as follows:

(1) Concussion: Transient brain dysfunction immediately after the head being hit by an external force. Clinically it manifests as transient coma, recent amnesia (retrograde amnesia), and symptoms including headache, nausea, or vomiting. No positive signs may be found during a neurologic examination.

(2) Cerebral contusion and laceration: Structural damage of brain tissues after head trauma. The injured brain tissue may show spotty or patchy bleeding, rupture, edema or necrosis, often accompanied by focal cerebral edema or diffuse brain swelling at the adjacent sites as well as varying degrees of intracranial hematoma.

(3) Intracranial hematoma: The most severe secondary lesions following brain injury. After brain injury, the blood may accumulate in some parts of the cranial cavity and increase intracranial pressure when it reaches a considerable volume, leading to a series of clinical symptoms. Based on the location, intracranial hematomas can be classified as epidural hematoma, subdural hematoma, and intracerebral hematoma.

(4) Brain stem injury: Primary or secondary brain stem injury following traumatic brain injury. Clinically the patient often falls into a coma.

(5) Spinal cord injury: The majority of spinal cord injuries originate from traffic accidents, falls, violence, or sports-related injuries. The severity of a spinal cord injury is determined by the degree of primary injury after the instantaneous force exerted on the spinal cord, which may cause spinal shock, non-complete injury, complete injury, and spinal cord transection. Common clinical manifestations of spinal cord injury include motor and sensory impairments below the level of injured spinal cord.

Registration Form for Death Cases

(Completed by a neurologist)

Name of survey site (district/county):___________	Code of survey site: □□□□□□	
Name of township/sub-district: ____________________________	Code of township/sub-district: □	
Name of village/residential community:_________	Code of village/residential community: □	
Code of household (same as on the preliminary screening form): □□□□	Code of the deceased family member (same as on the preliminary screening form): □	
Signature of neurologist:____________________<br>Date:	Signature of QC staff:___________________<br>Date:	
1. Name of deceased family member:___________	2. Sex:1=Male; 2=Female	└──┘
3. Date of death:		
4. Chief disease history, description of death process (including any predisposing factors, clinical manifestations, disease progression, and hospital where the diagnosis was made):		
____________________<br>____________________<br>____________________<br>____________________		
5. Diagnosis of the major fatal disease:		
5.1 Underlying disease: _________________________ICD code: └──┘-└──┘<br>5.2 Disease directly associated with death:_______________ICD code: └──┘-└──┘		
6.Hospital where the diagnosis was made:_________________________________		
7.Level of the hospital where the diagnosis was made:<br>1=Community hospital/township health care center/company clinic;<br>2=District/county hospital; 3=Provincial/municipal hospital or higher;<br>4=Did not visit a hospital; 99=Unknown		└──┘

8. Did the patient die from brain/spinal cord trauma?<br>1=Yes; 2=No (skip to question 9)	└───┘
9. Did the patient have brain/spinal cord trauma before death?<br>1=Yes; 2=No; 99=Unknown	└───┘

**Times of onset**	**Diagnosis**	**Date of onset**	**Locations**	**Main symptoms (multiple selection)**	**CT/MRI**	**Hospitalized**
First time	└───┘		└───┘	└───┘	└───┘	└───┘
Last time	└───┘		└───┘	└───┘	└───┘	└───┘

Aim: To determine the specific diagnosis following the brain/spinal cord trauma. If medical records or diagnosis documentation can be provided, record the diagnosis on these documents; if medical records or diagnosis documentation cannot be provided, base the diagnosis on the statement of the deceased person's family.

Diagnosis:

(1) Concussion: Transient brain dysfunction immediately after the head being hit by an external force. Clinically it manifests as transient coma, recent amnesia (retrograde amnesia), and symptoms including headache, nausea, or vomiting. No positive signs may be found during a neurologic examination.

(2) Cerebral contusion and laceration: Structural damage of brain tissues after head trauma. The injured brain tissue may show spotty or patchy bleeding, rupture, edema or necrosis, often accompanied by focal cerebral edema or diffuse brain swelling at the adjacent sites as well as varying degrees of intracranial hematoma.

(3) Intracranial hematoma: The most severe secondary lesions following brain injury. After brain injury, the blood may accumulate in some parts of the cranial cavity and increase intracranial pressure when it reaches a considerable volume, leading to a series of clinical symptoms. Based on the location, intracranial hematomas can be classified as epidural hematoma, subdural hematoma, and intracerebral hematoma.

(4) Brain stem injury: Primary or secondary brain stem injury following traumatic brain injury. Clinically the patient often falls into a coma.

(5) Spinal cord injury: The majority of spinal cord injuries originate from traffic accidents, falls, violence, or sports-related injuries. The severity of a spinal cord injury is determined by the degree of primary injury after the instantaneous force exerted on the spinal cord, which may cause spinal shock, non-complete injury, complete injury, and spinal cord transection. Common clinical manifestations of spinal cord injury include motor and sensory impairments below the level of injured spinal cord.

Location of injury: 1=Brain; 2=Spinal cord; 3= Both brain and spinal cord.

Main symptoms: 1=Unconsciousness or coma; 2=Cannot recall the cause or conditions of trauma after consciousness is recovered; 3=Paralysis or weakness of limbs or trunk; 4=Sensory disturbance of limbs

or trunk; 5=Fecal/urinary incontinence or retention; 99=Unknown

CT/MRI: 1=Yes; 2=No; 99=Unknown. **Hospitalized:** 1=Yes; 2=No; 99=Unknown.
